# Supplementary material for: Effects of Trichoderma harzianum combined with Phanerochaete chrysosporium on lignin degradation and humification during chicken manure and rice husk composting
Source: Front Microbiol. 2025 Feb 28;16:1515931. doi: 10.3389/fmicb.2025.1515931 (PMC11906335; doi:10.3389/fmicb.2025.1515931)
Supplement: Supplementary file 1 [file Data_Sheet_1.zip › 6.Function/All/FunGuild/l2/heatmap_group.pdf]

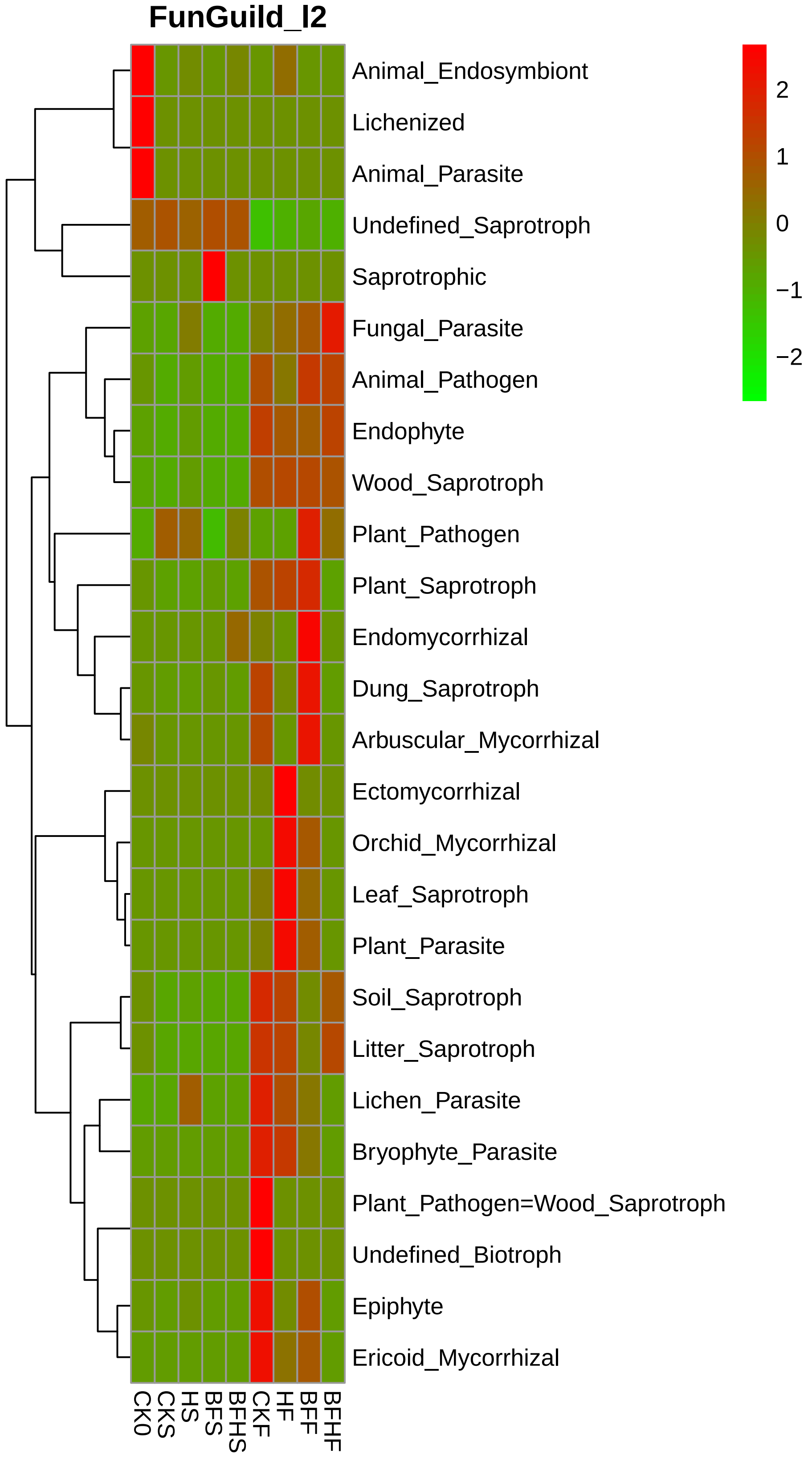
FunGuild_l2
Animal_Endosymbiont
Lichenized
Animal_Parasite
Undefined_Saprotroph
Saprotrophic
Fungal_Parasite
Animal_Pathogen
Endophyte
Wood_Saprotroph
Plant_Pathogen
Plant_Saprotroph
Endomycorrhizal
Dung_Saprotroph
Arbuscular_Mycorrhizal
Ectomycorrhizal
Orchid_Mycorrhizal
Leaf_Saprotroph
Plant_Parasite
Soil_Saprotroph
Litter_Saprotroph
Lichen_Parasite
Bryophyte_Parasite
Plant_Pathogen=Wood_Saprotroph
Undefined_Biotroph
Epiphyte
Ericoid_Mycorrhizal
CK0
CKS
HS
BFS
BFHS
CKF
HF
BFF
BFHF
2
1
0
−1
−2
